# Anti-HER2 therapy in metastatic breast cancer: many choices and future directions

**DOI:** 10.1007/s10555-022-10021-x

**Published:** 2022-02-10

**Authors:** Carrie S. Wynn, Shou-Ching Tang

**Affiliations:** Cancer Center and Research Institute, University of Mississippi Medical Center, Guyton Research Building, G-651-07, 2500 North State Street, Jackson, MS 39216 USA

**Keywords:** Anti-HER2 therapy, HER2 + metastatic breast cancer, Treatment, Drug resistance, New drugs

## Abstract

Metastatic HER2 + breast cancer is an expanding area of drug development and research, with three new drugs approved in 2020 alone. While first-line therapy is well-established for metastatic HER2 + breast cancer, the standard of care for second-line therapy will likely be changing soon based on the results of the DESTINY-Breast03 trial. In the third-line setting, many options are available. Considerations in choosing between regimens in the third-line include resistance to trastuzumab, the presence of brain metastases, and tolerability. High rates of resistance exist in this setting particularly due to expression of p95, a truncated form of HER2 that constitutively activates downstream signaling pathways. We suggest a tyrosine kinase inhibitor (TKI)-based regimen because of the activity of TKIs in brain metastases and in p95-expressing tumors. Attempts to overcome resistance to anti-HER2 therapies with PI3K inhibitors, mTOR inhibitors, and CDK 4/6 inhibitors are an active area of research. In the future, biomarkers are needed to help predict which therapies patients may benefit from the most. We review the many new drugs in development, including those with novel mechanisms of action.

## Introduction

Approximately 15–20% of breast cancers overexpress HER2, a tyrosine kinase receptor that is a member of the epidermal growth factor receptor (EGFR) family [1, 2]. Overexpression of HER2 triggers multiple downstream pathways that enhance proliferation of cancer cells [3]. Since levels of HER2 strongly correlate with carcinogenesis, it is considered an adverse prognostic marker [4] and is associated with increased resistance to chemotherapeutic agents [5]. However, normal adult tissue cells do not express much HER2 and are less sensitive to HER2-targeting agents, making HER2 an ideal target for cancer treatment [4].

There are multiple anti-HER2 therapies currently approved by the FDA, many of which have emerged within the last 2 years. It is becoming increasingly difficult for clinicians in busy practices to decide on which particular anti-HER2 therapy to use, especially in the late-line treatment of metastatic disease. We present this review to highlight these novel agents, including their mechanisms of action, efficacy and toxicities, and the rationale in choosing between particular agents in each line of therapy. We also discuss the future development of anti-HER2 therapy in metastatic breast cancer (MBC).

## Current anti-HER2 therapies by mechanism of action

### Monoclonal antibodies

There are three monoclonal antibodies against HER2 that are currently approved: trastuzumab, pertuzumab, and margetuximab. Trastuzumab and pertuzumab bind to different extracellular domains of the HER2 receptor and thus have complementary mechanisms of action. Though pertuzumab alone has shown only modest clinical antitumor activity, it has a synergistic effect when combined with trastuzumab [6]. Margetuximab is a recently approved chimeric antibody that shares epitope specificity with trastuzumab and also incorporates an engineered Fc region to increase immune activation against HER2 [7]. This engineered Fc receptor binds with higher affinity to the stimulatory CD16A receptor found on macrophages and natural killer cells [8]. There are multiple proposed theories for how monoclonal antibodies exert an antitumor effect, including inducing antibody-dependent cell-mediated cytotoxicity (ADCC), inhibiting downstream signal transduction pathways (such as PI3K), and interfering with DNA repair, among others [4].

### Tyrosine kinase inhibitors

Tyrosine kinase inhibitors (TKIs) bind to the adenosine triphosphate (ATP)-binding domain of EGFR receptors, which inhibits tyrosine kinase phosphorylation and suppresses downstream signaling [9]. Several TKIs are available on the market today, including lapatinib, neratinib, and most recently tucatinib [2, 9]. Lapatinib reversibly binds HER1 (also known as EGFR) and HER2. Neratinib is a second-generation TKI that has irreversible pan-HER activity [9]. Tucatinib is a potent, highly selective inhibitor of the kinase domain of HER2 and minimally inhibits the EGFR receptor, which in theory should decrease toxicity [2]. Advantages of TKIs include oral administration and better blood–brain barrier penetration [9]. There is also less cardiac toxicity, and patients who developed congestive heart failure from trastuzumab may safely be treated with lapatinib [10].

### Antibody–drug conjugates

Antibody–drug conjugates (ADCs) are monoclonal antibodies that are connected to a cytotoxic agent with a linker. Because of the specificity of the antibodies and because of the limited number of molecules that enter the cell, the potency of cytotoxic drugs used with ADCs are usually much higher than with traditional chemotherapy [11]. Currently available ADCs for HER2 + breast cancer include ado-trastuzumab emtansine (T-DM1) and trastuzumab deruxtecan (T-DXd). For T-DM1, trastuzumab is conjugated to a microtubule inhibitor called DM1, a derivative of maytansine [12]. The cytotoxic agent of T-DXd is a topoisomerase I inhibitor. Compared with T-DM1, T-DXd has a higher drug-to-antibody ratio (8 versus 3.5). The payload of T-DXd easily crosses the cell membrane and has a short half-life to make it more potent while minimizing systemic exposure [13].

**Fig. 1:**
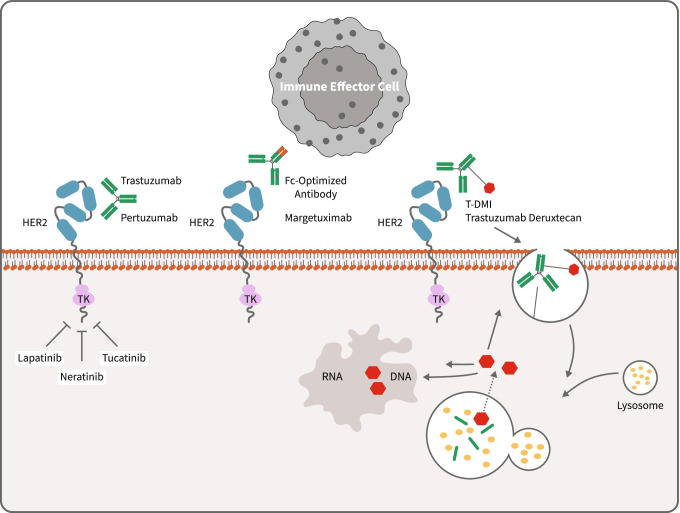
Mechanisms of action of current anti-HER2 therapies. Mechanisms of action of anti-HER2 therapy by monoclonal antibodies (trastuzumab and pertuzumab), Fc-optimized antibody (margetuximab), tyrosine kinase inhibitors (lapatinib, neratinib, and tucatinib), and antibody–drug conjugate (ado-trastuzumab emtansine and trastuzumab-deruxtecan)

Mechanisms of action of available anti-HER2 therapies are summarized in Fig. **1**.

## Currently available options: first- and second-line

### HR + /HER2 + and indolent disease

Evidence suggests that patients without visceral crisis or rapidly progressive disease can avoid up-front chemotherapy. Based on preclinical evidence, crosstalk between HER2- and hormone receptor (HR)-signaling pathways may contribute to resistance to endocrine therapy (ET), and by blocking HER2, ET may become more effective [14]. Options include ET + trastuzumab [15], lapatinib + letrozole [16], and dual anti-HER2 therapy with trastuzumab + lapatinib + an aromatase inhibitor (AI) [14], the latter of which may be preferable in women who have received prior trastuzumab.

### ER − or ER + with visceral crisis or hormone refractory

For patients who do have rapidly progressive disease or visceral crisis, chemotherapy is generally required to obtain disease control. For first-line treatment, docetaxel + trastuzumab + pertuzumab (THP) is preferred based on the results of the CLEOPATRA trial, in which the addition of pertuzumab to the backbone of docetaxel + trastuzumab significantly improved PFS and OS in previously untreated HER2 + MBC [17]. As of today, T-DM1 is the preferred second-line treatment per the EMILIA trial, in which it improved PFS and OS with less toxicity compared with lapatinib + capecitabine [12]. However, trastuzumab deruxtecan will likely replace T-DM1 in this setting based on the results of the DESTINY-Breast03 trial, which was recently presented at the European Society for Medical Oncology (ESMO) Congress 2021 meeting. In this trial, T-DXd was compared with T-DM1 in the second-line setting in patients with HER2 + MBC who had progressed on a taxane and trastuzumab. At 16 months, median PFS was not reached with T-DXd but was 6.8 months with T-DM1 (HR = 0.28; *P* = 7.8 × 10^−22^) [18].

T-DM1 can also be used in the first-line setting for patients who cannot tolerate THP, as it is less toxic and patients maintain health-related quality of life (HRQOL) longer [19].

## Third-line treatment: many options

In the third-line setting, multiple options are now available, and there is no standard of care. We review these based on mechanism of action.

### TKIs

Prior to the development of neratinib and tucatinib, lapatinib + capecitabine was commonly used in the late-line setting based on its superiority over capecitabine alone in women who had progressed on standard chemotherapy and trastuzumab [20]. More recently, in the NALA trial, lapatinib was compared head-to-head with neratinib (both with capecitabine) in patients with metastatic disease who had received at least two prior anti-HER2 therapies. The ORR (32.8% v 26.7%), clinical benefit rate (44.5% v 35.6%), and median duration of response (8.5 v 5.6 months) were all higher in the neratinib group, as was PFS (8.8 vs 6.6 months; HR 0.76; 95% CI, 0.63 to 0.93; *P* = 0.003). Notably, fewer patients in the neratinib arm required intervention for CNS disease (cumulative incidence, 22.8% vs 29.2%; *P* = 0.043), suggesting either prevention or delayed time to development. As might be expected, grade 3 diarrhea was more common in the neratinib group (24.4% of patients) despite using a lower dose of capecitabine and mandatory prophylactic antidiarrheals. However, quality of life scores remained similar between the two groups [21].

A tucatinib-based regimen is recommended for patients with visceral and brain metastases who progress on T-DM1 per NCCN guidelines [22] based on the results of the HER2CLIMB trial. In this study, heavily pretreated patients were randomized to receive capecitabine + trastuzumab ± tucatinib. PFS at 1 year was 33.1% in the tucatinib group vs 12.3% with placebo (HR for disease progression or death, 0.54; 95% CI, 0.42 to 0.71; *P* < 0.001). At 2 years, OS was 44.9% with tucatinib, compared with 26.6% in the placebo arm (HR for death, 0.66; 95% CI, 0.50 to 0.88, *P* = 0.005) [2]. For the subgroup with brain metastases, the risk of intracranial progression or death was decreased by 68% (HR, 0.32; 95% CI, 0.22 to 0.48; *P* < 0.0001) [23]. Though there were more side effects in the tucatinib group, < 6% of patients discontinued treatment due to adverse effects [2].

### Monoclonal antibodies

Trastuzumab plus chemotherapy has been evaluated in multiple phase II studies of HER2 + MBC that has progressed on trastuzumab. Chemotherapies studied include capecitabine [24], paclitaxel [25], docetaxel [26], vinorelbine [27], gemcitabine [28], and eribulin [29]. These regimens appear to have similar efficacy, with response rates ranging from 20–30% and PFS 3–8 months, though the activity in patients pretreated with pertuzumab or T-DM1 is unknown. These regimens mainly differ in their toxicity profiles, which are reflective of their chemotherapy partners.

The FDA also recently approved margetuximab in combination with chemotherapy in the third-line setting (SOPHIA trial). In this study, patients received single-agent chemotherapy plus either margetuximab or trastuzumab. In the overall population, margetuximab was associated with a small (but statistically significant) prolongation of PFS by approximately 1 month (4.9 vs 5.8 months, HR 0.76; *P* = 0.03) [7], which was the basis for the FDA approval. However, the results of the final OS analysis were recently announced, in which there was no difference between the groups [30]. SOPHIA was the first prospective trial to investigate the impact of Fc-gamma receptor alleles. Patients with the low-affinity CD16A-158F allele (82% of study patients) benefitted more from margetuximab both in terms of PFS (6.9 vs 5.1 months; HR, 0.68) [31] and OS (23.3 months vs 20.8 months; HR = 0.86) [30].

### Antibody–drug conjugates

Trastuzumab deruxtecan was approved after the single-arm phase II DESTINY-Breast01 trial, in which heavily pretreated women with HER2 + MBC were treated with T-DXd. Results were impressive. Despite a median number lines of prior therapy of 6, the ORR was 60.9% (95% CI, 53.4 to 68.0), and the median duration of response was 14.8 months (95% CI, 13.8 to 16.9). However, concerns arose because of the relatively high rates of interstitial lung disease (ILD) in this study (13.6%), from which four patients died. ILD was reported as a late complication of treatment, with a median time to onset of 193 days [13]. Rates of ILD in other studies of T-DXd have somewhat differed. A combined analysis of all patients treated with T-DXd in phase I and II studies found that 15.5% of patients experienced drug-related ILD. Though most cases were mild, 6 patients (2.4%) had grade 5 ILD. Interestingly, in this analysis the median time to onset of ILD was much earlier at 5.6 months, but after 12 months the risk was low [32]. In the DESTINY-Breast03 trial, the rate of interstitial lung disease was lower than in previous trials at 10.5%, with no grade 4/5 ILD [18]. The lower rates of ILD in this study may be related to less exposure to prior chemotherapy. There has also been increased education and awareness about ILD and T-DXd. NCCN guidelines currently recommend T-DXd as the preferred agent for patients with metastatic disease who have progressed on T-DM1 [22]. As discussed above, trastuzumab deruxtecan will likely become the preferred agent in the second-line once the results of DESTINY-Breast03 are published. A phase III study of trastuzumab deruxtecan versus physician’s choice in the third-line setting is also ongoing (DESTINY-Breast02, NCT03523585), as is another phase III study looking at its activity in metastatic tumors that are HER2-low (DESTINY-Breast04, NCT03734029).

### Non-chemotherapy anti-HER2 therapy

For those with HR + /HER2 + tumors and indolent disease, trastuzumab or lapatinib in combination with hormonal therapy is an option if the patients have not been previously exposed to these agents. For those with HR − /HER2 + tumors and indolent disease, or for patients who do not want or cannot tolerate chemotherapy, “biologics-only” with trastuzumab and lapatinib is a viable option (dual-HER2 blockade) based on data from early-stage disease that suggests synergy between the two [33, 34].

## Fourth-line and beyond

In the fourth-line and beyond setting, patients can continue anti-HER2 therapy with either a TKI or a monoclonal antibody + single-agent chemotherapy. It is likely that dual-HER2 blockade can be used with other newer anti-HER2 TKIs such as neratinib or tucatinib and anti-HER2 monoclonal antibodies like margetuximab, although we do not yet have evidence to demonstrate their efficacy and safety.

**Fig. 2:**
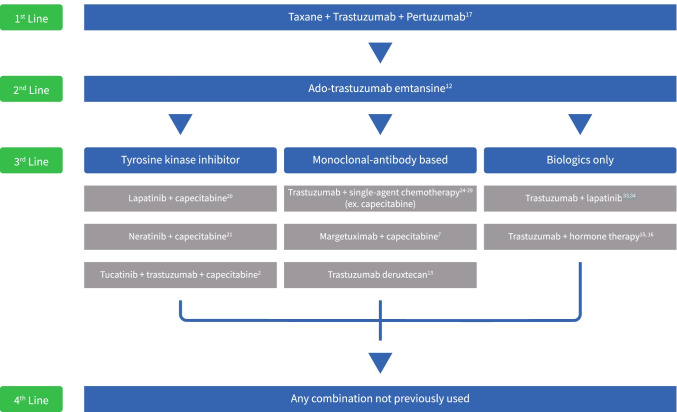
Current treatment algorithm for anti-HER2 therapy in the metastatic setting

A summary of current treatment options is depicted in Fig. 2.

Major results from clinical trials are summarized in Table 1.

**Table 1:** **Currently available anti-HER2 therapy in third-line setting**. Summary of major results from clinical trials

	Lapatinib + capecitabine^20,115^ (*n* = 163)	NALA: Neratinib + capecitabine^21^ (*n* = 307)	HER2CLIMB: Trastuzumab + capecitabine + tucatinib^2^ (*n* = 320)	Trastuzumab + capecitabine^24^ (*n* = 56)	SOPHIA: Margetuximab + single-agent chemotherapy^7,30^ (*n* = 266)	DESTINY-Breast01: Trastuzumab deruxtecan^13^ (*n* = 184)
RR, no. (%)	35 (22)	84 (32.8) (*n* = 256)	138 (40.6)	28 (50)	67 (25.2)	112 (60.9)
Median PFS (months)	8.4	5.6	7.8	9.2**	5.7	16.4
Median OS (months)	17.3 (*n* = 207)	24.0*	21.9	25.6	21.6	NR

^*^Reported as mean OS

^**^Reported as time to progression (TTP) rather than PFS

Abbreviations: *RR* response rate, *PFS* progression free survival, *OS* overall survival, *NR*, not reached

## Resistance to anti-HER2 therapy

Despite these major advances in HER2 + MBC, most patients will ultimately die of their disease. Less than 30% of HER2-positive patients respond to trastuzumab monotherapy [35], and another 70% who initially respond will progress to metastatic disease within a year. This suggests both innate and acquired resistance mechanisms [36]. Understanding the mechanisms of resistance to HER2-targeted treatments will be essential in developing new therapeutic strategies and improving survival.

### p95

One known mechanism of resistance to trastuzumab is the expression of a truncated version of HER2 called p95, named after its molecular weight. This HER2 lacks the extracellular domain to which trastuzumab binds and constitutively activates downstream signaling through its intracellular tyrosine kinase domains. The prevalence of p95 expression in HER2 + BC appears to be around 30% [37]. High levels of p95 expression correlate with increased nodal metastasis [38] and lower 5-year disease-free survival [39]. All trastuzumab-based therapy, including margetuximab, is unlikely to be effective in HER2 + breast cancers with p95 expression due to the lack of an extracellular trastuzumab-binding domain. A TKI-based regimen is likely the best choice for these tumors. Studies have shown that though p95 tumors do not respond to trastuzumab [5], lapatinib is equally as effective in tumors expressing p95 as those without [40]. Furthermore, a PFS benefit favoring neratinib + capecitabine over lapatinib + capecitabine in high p95 tumors was observed in a biomarker analysis of p95 tumors in the NALA trial [41]. Other splice variants of HER2 have been identified, including p100, Δ16-HER2, and Herstatin. Though less is known about these variants, these can also alter response to anti-HER2 treatment [42].

### PI3K/AKT/mTOR pathway

Other mechanisms of resistance to anti-HER2 therapies have been described, most of which involve activating alternative (such as IGF-1R) or downstream signaling pathways [5, 43]. The most clinically important of these appears to be the PI3K/AKT/mTOR pathway. The PI3K pathway can be activated by loss of PTEN expression, mutations in PIK3CA, and amplification of AKT [44, 45]. PIK3CA mutations are present in about 20% of HER2 + breast cancers [46]. Activation of the PI3K pathway is associated with resistance to trastuzumab [47]. Patients with both loss of PTEN and PIK3CA mutations have a significantly shorter PFS and OS after trastuzumab therapy than those without [44, 45], and in the neoadjuvant setting, patients with PI3K mutations are less likely to achieve a pathologic complete response even when dual anti-HER2 therapy is used [46]. This suggests that the PI3K pathway is a major determinant of resistance to trastuzumab [44].

In light of this, the use of mTOR inhibitors and PI3K inhibitors to overcome resistance to current HER2 therapies is an active area of research [43]. In the BOLERO-3 trial, the addition of everolimus (an mTOR inhibitor) to chemotherapy + trastuzumab improved PFS from 5.8 to 7 months in patients with trastuzumab-resistant HER2 + MBC. Patients with loss of PTEN expression appeared to derive more benefit than those without [48]. This data supports the idea that mTOR inhibition may help overcome trastuzumab resistance, though where this fits in the context of other therapies is not currently defined. As for PI3K inhibition, a phase III clinical trial is ongoing evaluating alpelisib in combination with trastuzumab and pertuzumab following induction with THP for patients with HER2 + MBC and PI3K mutations (NCT04208178). ADCs may play a role as well in PI3K-mutated tumors. In an exploratory analysis of the EMILIA trial, in the T-DM1 group, there was no difference in outcomes based on PI3K mutational status, suggesting that T-DM1 may be effective for PIK3CA-mutated tumors [49].

### Cyclin D1/CDK4/CDK6

In addition to the PI3K/AKT/mTOR pathway, previous work in mouse models has demonstrated that cyclin D1/CDK4 can mediate resistance to HER2 therapy and that by inhibiting CDK4/6, tumors can be re-sensitized to anti-HER2 treatments [50]. The PATRICIA trial combined palbociclib with trastuzumab in postmenopausal women with HER2 + MBC who had received 2–4 lines of anti-HER2 therapy. Interim analysis showed a marked difference in PFS between patients with luminal versus non-luminal disease (10.6 vs 4.2 months). These results were intriguing in that they not only demonstrated a benefit for the addition of a CKD4/6 inhibitor, but also suggested a specific biomarker to predict the patients who would benefit [51].

### Genetic heterogeneity

Another challenge that arises with HER2 + breast cancer is the issue of intra- and intertumoral heterogeneity. Since the advent of trastuzumab, guidelines published by the American Society of Clinical Oncology (ASCO) and College of American Pathologists (CAP) have attempted to optimize thresholds for defining HER2 positivity [52]. Several patterns of HER2 expression have emerged, leading to the term “HER2 heterogeneity” [53]. HER2 status may differ between cells within the same tumor, known as intratumoral heterogeneity, or may differ between the primary tumor and its metastases, so-called intertumoral heterogeneity. HER2 heterogeneity is significantly more common in HER2 equivocal cases, and frequencies as high as 40% have been described [54].

Clinically, HER2 heterogeneity has been associated with larger size, higher grade histology, increased frequency of lymph node metastasis, and shorter disease-free and overall survival. Notably, patients appear to be less responsive to anti-HER2 therapy [53]. Filho et al. recently prospectively evaluated this in a phase II clinical trial, in which patients with HER2 + BC were treated with T-DM1 and pertuzumab prior to surgery. No patients with heterogeneous tumors achieved a pathologic complete response (pCR), while the pCR rate was 55% in those without heterogeneous tumors [55].

### HER2 low

One exciting development is the activity of newer anti-HER2 agents in HER2-low disease, which typically is defined as those with a HER2 immunohistochemistry (IHC) score of 1 + or 2 + with negative *in* *situ* hybridization (ISH). More than half of patients with breast cancer may qualify as HER2-low [56]. Though previously HER2-targeted agents (including T-DM1) have not been effective in this subset of patients, results with novel ADCs are encouraging. In a phase I study, T-DXd produced a response rate of 37% in heavily pre-treated patients with HER2-low MBC, and the median duration of response was 10.4 months [57]. Similarly, a partial response was achieved in 28–40% (depending on ER status) of patients with HER2-low disease treated with trastuzumab duocarmazine [58]. The presence of cleavable linkers and higher membrane permeability likely accounts for the activity of the newer ADCs in HER2-low disease, as they produce a prominent bystander effect on surrounding non-antigen-expressing cancer cells. The activity of T-DXd may be independent of HER2 expression. Other therapies, such as vaccines and bispecific antibodies, have been tested in HER2-low disease as well with promising results. Defining HER2-low remains a challenge, as the current methods of HER2 testing may not be adequate to identify tumors with low levels of HER2 expression that could benefit from these newer therapies [56].

### Activating HER2 mutations without copy number gain

Even in the presence of a normal HER2 gene copy number, HER2 signaling can be activated by somatic mutations, most frequently in the tyrosine kinase domains [59]. In breast cancer, the frequency of these mutations is about 2–5%, though in a study of patients with highly pretreated metastatic disease and high tumor burden, the frequency was 8.9%. This and other literature suggests that these mutations may be induced by anti-cancer therapies [60]. HER2 mutations may be enriched in certain histological subtypes such as invasive high-grade lobular carcinomas [61]. These mutations may affect sensitivity to anti-HER2 therapy by activating oncogenic signaling pathways independent of drug-target binding [59]. Somatic mutations also appear to confer resistance to endocrine therapy in HR + MBC [62]. However, many of these mutations are actionable and represent a HER2-targeting opportunity even in HER2-negative breast cancers. Preclinical work suggested that many of these mutations are sensitive to neratinib (but not lapatinib) [63], and subsequently several clinical trials have demonstrated a benefit with neratinib in patients with HER2-mutated nonamplified MBC [62, 64, 65]. Notably in the SUMMIT trial, neratinib demonstrated activity in all types and classes of HER2 mutations in breast cancer [65].

### Overexpression of HER1 and HER3

The HER2 receptor is unique in that it does not have a ligand, but instead is activated by either heterodimerization with other members of the EGFR family (particularly EGFR and HER3) or homodimerization with itself when HER2 is expressed at very high levels [66]. Overexpression of EGFR and HER3 can activate HER2 cell signaling pathways (such as PI3K) and lead to tumor proliferation [67, 68]. Evidence suggests that overexpression of EGFR is a negative predictor of pathologic complete response and is associated with decreased overall survival. Similarly, patients with high HER3 expression levels have been shown to have shorter PFS and OS compared with patients with low HER3 expression. Targeting EGFR with EGFR inhibitors, though promising in pre-clinical studies, has not proven to be efficacious in clinical trials [68]. Both neratinib and lapatinib have shown activity in EGFR-amplified breast cancer cell lines. The irreversible, pan-HER activity of neratinib may ultimately make neratinib more effective in this population, because the more selective inhibition of HER receptors by lapatinib could allow resistance to develop through activation of other HER family receptors [69].

### Reduced expression of HER2

One final mechanism of resistance to trastuzumab-based therapy is decreased expression of HER2, which has been detected in a wide range of T-DM1-resistant cell lines [70], and has been associated with reduced rates of pCR and poor recurrence-free survival [71].

### Biomarker testing

**Fig. 3:**
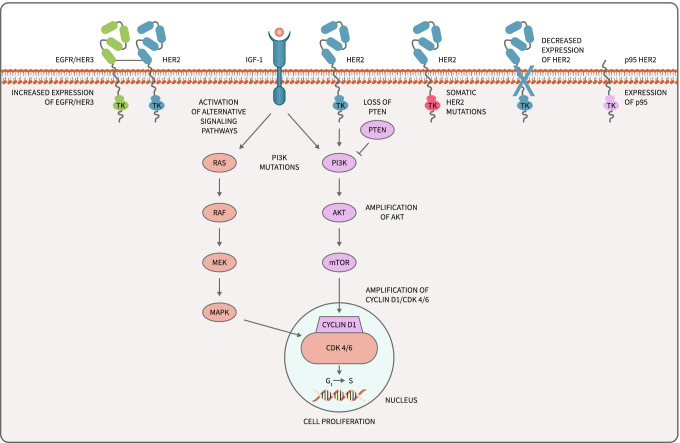
Pathways of resistance to anti-HER2 therapies, including p95. Important pathways involved in anti-HER2 resistance such as the PI3K/AKT/mTOR pathway and the expression of HER2 p95 lacking the extracellular trastuzumab-binding domain

In the era of precision oncology, the development of biomarkers to predict response to anti-HER2 therapy is urgently needed to both improve outcomes and reduce toxicity. Tumor heterogeneity and the complexity of drug resistance mechanisms make this research challenging [43]. It is currently unknown if p95 or other biomarkers may be used to guide clinicians to choose one anti-HER2 therapy over another. One promising area of biomarker testing is the role of circulating tumor DNA (ctDNA), which is currently being studied in a large number of solid tumors. Evidence for the use of ctDNA in breast cancer is rapidly evolving. Though tissue analysis is the gold standard for identifying tumor mutations, this is invasive and not practical for serial monitoring to identify mutations that may be acquired through treatment. Tissue analysis may also fail to capture intra- and intertumor heterogeneity [72]. ctDNA allows for sensitive and specific serial testing over time, is safer and less expensive, and can overcome issues with tumor heterogeneity [73, 74]. Changes in ctDNA levels also correlate with changes in tumor burden, making it a sensitive biomarker for monitoring tumor response [74, 75]. In breast cancer, ctDNA can be used to detect somatic HER2 [60], PI3K, PTEN, and AKT mutations, among others [76, 77]. Loss of HER2 expression has also been detected in ctDNA and was associated with resistance to T-DM1 [70]. Issues remain, however. ctDNA assays may fail to capture mutations detected by genotyping tumor specimens, and the majority of ctDNA assays in advanced cancer have insufficient evidence to guide their use in clinical practice [78]. Further research is needed to clarify the role of ctDNA in the clinic (Fig. 3).

## Choice of therapy in the third-line setting

Although there is no standard anti-HER2 therapy to choose in the third-line setting, two principles of drug sequencing still apply: 1) To avoid cross-resistance, drugs used in later lines of therapy should have different mechanisms of action than those used previously, and 2) there should be no overlapping toxicities. In the third-line setting, there are three reasons to consider a TKI as one’s first choice: development of resistance to trastuzumab-based therapy, to prevent or treat brain metastases, or to offer patients a break from intravenous chemotherapy.

### Resistance to trastuzumab-based therapy

As discussed previously, resistance to trastuzumab-based therapy is common in the late metastatic setting. In the absence of brain metastases, neratinib + capecitabine is an excellent choice in the third line, based on the results of the NALA trial and from other studies mentioned above which showed a benefit with neratinib in tumors with resistance to trastuzumab. Though high rates of grade 3 diarrhea have traditionally made neratinib difficult to tolerate, this can be significantly offset with the use of a dose-escalated regimen [79].

### Brain metastases

Between 30 and 55% of patients with HER2 + MBC will develop brain metastases at some point in the course of their disease [21]. The efficacy of systemic therapies is usually limited by inability to penetrate the blood–brain barrier [80]. Historically, patients with brain metastases have often been excluded from clinical trials. However, TKIs are smaller molecules known to have good blood–brain barrier penetration, and all three have shown efficacy in patients with CNS disease [21, 23, 81]. Among the TKIs, a tucatinib-based regimen has emerged as the preferred therapy for patients with brain metastases based on the HER2CLIMB trial, which was one of the first randomized controlled trials to demonstrate a meaningful increase in OS (> 6 months) in patients with brain metastases. In this study, among the 174 patients with active brain metastases, the 1-year CNS-PFS was 35% in the tucatinib arm versus 0% in the control arm (HR 0.36, *P* < 0.001), with a confirmed intracranial ORR of 47.3%. A small number of patients (44 in the tucatinib arm) with active, untreated brain metastases elected to delay radiation therapy in favor of systemic therapy and still achieved a median CNS-PFS of 8.1 months, suggesting that tucatinib could delay the need for radiation. In patients who continued on trial after local management with radiation, tucatinib appeared to delay subsequent disease progression [23].

There is also evidence that ADCs have activity in brain metastases. This was first described with T-DM1 in case reports and smaller studies [11]. A post hoc analysis of the KAMILLA trial showed that in patients with measurable brain metastases treated with T-DM1, almost half had stable disease or better for at least 6 months (CBR 42.9%, 95% CI 34.1–52.0) [82]. In a retrospective exploratory analysis of the EMILIA trial, PFS was similar between the T-DM1 and capecitabine-lapatinib arms (5.9 vs 5.7 months, HR = 1.00) in the subgroup of patients with treated, asymptomatic CNS metastases at baseline. There was also a significant difference in OS favoring the T-DM1 group (26.8 months vs 12.9 months, HR 0.38, *P* = 0.008) [80]. Trastuzumab deruxtecan has also demonstrated activity in brain metastases. In the DESTINY-Breast01 trial, the 24 patients with CNS disease had an ORR of 58.3% and a median PFS of 18.1 months, which was comparable to the results of the total patient population [83]. The activity of T-DXd in brain metastases is currently being evaluated in the phase II TUXEDO-1 trial. Thus far, 5/6 (83.3%) patients have had an intracranial response [84]. Presumably, the disrupted blood–brain barrier in patients with brain metastases enables ADCs to have activity there, where previously these molecules were too large to cross. The other possibility is the free deruxtecan cleaved off from the ADC in the circulation can easily penetrate the blood brain barrier.

## Issues with current treatment options

### ADCs

The hope for ADCs was that they would be the “magic bullet” that could deliver higher-dose cytotoxic chemotherapy directly to target cells without the effects on non-target tissue. However, this has not been borne out in clinical practice, where a substantial number of side effects have been observed. Most side effects of ADCs are related to payload effects in off-target tissues, reflecting either cleavable peptide linkers prematurely releasing the drug into the bloodstream or a prominent bystander effect [11]. For instance, the incidence and severity of the systemic toxicities of trastuzumab deruxtecan, including ILD, neutropenia, alopecia, etc., are similar to other topoisomerase inhibitors like topotecan and irinotecan. Trastuzumab deruxtecan uses a cleavable tetrapeptide linker, which is more likely to release the payload before the ADC reaches the target tumor cells. This may explain its activities in tumors that are low in HER2 expression and in tumors with brain metastasis. On the other hand, for ADCs with noncleavable linkers such as T-DM1, the off-target systemic side effects are likely from the lysed tumor cells releasing the free payload. Indeed, we recently reported that the systemic toxicities of T-DM1 highly correlate with anti-tumor efficacy and patient survival [85].

### Margetuximab

As for margetuximab, the results from the SOPHIA trial represent a further step towards personalization, as there was a small PFS and OS benefit for patients with the low-affinity CD16A allele, but none for those without. However, the drug was approved for use without testing for the CD16A polymorphism (which is difficult to test); thus, patients who may not benefit from the drug may still receive it.

## Future directions

Current research in anti-HER2 therapy is focusing on developing novel TKIs, ADCs, bispecific antibodies, CAR-T, immunotherapy, and inhibiting protein production or increasing degradation.

### Novel TKIs

Numerous treatments are currently on the horizon for HER2 + MBC. Pyrotinib is an irreversible pan-HER receptor TKI that targets EGFR, HER2, and HER4. In the PHOEBE trial, patients with HER2 + MBC who had previously been treated with trastuzumab and taxanes were randomized to capecitabine plus either pyrotinib or lapatinib. PFS was nearly doubled in the pyrotinib group (12.5 versus 6.8 months), though at a cost of increased toxicity [86]. Pyrotinib is not currently approved in the USA but is approved in China for use with capecitabine in patients with HER2 + MBC who have progressed on anthracyclines or taxanes [87].

### ADCs

Several new ADCs are currently under investigation. (Vic-)trastuzumab duocarmazine (SYD985) is a new ADC that has shown promising results in early clinical trials and was granted fast-track recognition by the FDA. The payload of SYD985 is an alkylating agent that is attached to trastuzumab by a stable linker [88]. In preliminary studies, the ORR was 33%, with a median PFS of 9.4 months. Eighty percent of these patients had previously progressed on T-DM1. SYD985 has also shown activity in HER2-low and triple-negative breast cancer. Fatigue, conjunctivitis, dry eyes, and increased lacrimation were the most common adverse effects [89]. The results of the phase III TULIP study (NCT02277717) will likely be announced within the next year.

Trophoblast cell-surface antigen-2 (Trop-2) is a tumor-associated calcium signal transducer that stimulates cancer growth [90] and is expressed in all types of breast cancer, including HER2 + disease [91], as well as a variety of other solid tumors. High levels are associated with a worse prognosis [92]. Sacituzumab govitecan is an anti-Trop2-SN-38 ADC that is approved for triple negative breast cancer [93] and has also shown promising results in HR + /HER2- MBC [94]. Future studies will need to determine its role in HER2 + MBC, though likely it will have activity there as well.

### Bispecific antibodies

ZW25 is a bispecific antibody that simultaneously binds extracellular domain (ECD) 4, the trastuzumab domain, and ECD2, the pertuzumab domain, of HER2. In a phase I study, patients with heavily pre-treated HER2 + MBC had a response rate of 33% and was remarkably well tolerated, with no treatment discontinuations due to adverse effects [95]. A phase II clinical trial is currently underway evaluating ZW25 in combination with palbociclib and fulvestrant, with the goal of finding another chemotherapy-free option for patients with advanced HR + /HER2 + breast cancer (NCT04224272).

### Bispecific T-cell engagers (BiTE)

PRS-343 is bispecific T-cell engager (BiTE) that promotes binding of CD137 + (a key costimulatory immunoreceptor) T-cells to HER2 + tumor cells, thereby enhancing local immune activation and decreasing peripheral toxicity. PRS-343 has currently entered a phase I clinical trial, where it has shown promising results, with a disease control rate of 58% and no grade 3 or 4 adverse effects [96].

### Biparatopic ADCs

ZW49 is a biparatopic ADC, an innovative therapy that combines the technology of bispecific antibodies with ADCs. ZW25 acts as the targeting agent, allowing for enhanced delivery of the payload to cancer cells. The payload of ZW49 is a novel agent called N-acyl sulfonamide auristatin. Preclinical results were promising, and a phase I clinical trial is now ongoing [97].

### CAR-T against HER2

Though chimeric antigen receptor (CAR)-T has had promising results in hematologic malignancies, it has not been as effective in treating solid tumors. However, recent studies are promising for the use of CAR-T in HER2 + MBC, particularly when anti-HER2 CAR-T cells are combined with PD-1 antibodies [98]. Clinical trials are currently underway evaluating CAR-T in HER2 + breast cancer with brain or leptomeningeal metastases (NCT03696030). Of note, there is a case report of a woman with metastatic HER2 + colon cancer who underwent CAR-T and developed respiratory distress within 15 min of her infusion and ultimately died 5 days later. Laboratory analysis was consistent with cytokine storm. Authors speculated that the anti-HER2 T cells localized to the lungs immediately after infusion and were triggered to release inflammatory cytokines by the low levels of HER2 present on normal lung cells [99]. However, the low levels of HER2 expressed on normal lung tissue do not likely explain the lung toxicity seen with T-DXd, as lung toxicity is uncommon with other HER2-directed therapies, including trastuzumab, pertuzumab, T-DM1, and the anti-HER2 TKIs. Topoisoerase I inhibitors such as irinotecan cause interstitial lung disease. Free payload of deruxtecan from the ADC may similarly result in the lung toxicity.

### Agents that inhibit HER2 protein production or induce its destruction

Aptamers are synthetic single-stranded oligonucleotides that can be crafted to bind to specific target proteins on the cell surface, which then induces endocytosis. This can be exploited to deliver therapeutic cargoes such as small interfering RNA (siRNA) [100], which can silence sequence-specific genes. The specificity of these aptamer-siRNA chimera (AsiC) enables them to be immunogenic while minimizing off-target tissue side effects, a concept similar to ADCs. Making the aptamers bivalent via an antibody-like structure facilitates the engagement of cell-surface proteins and significantly increases the amount of siRNA delivered [101]. Several anti-HER2/3 aptamers are in development. We have developed a bivalent HER2 aptamer-EGFR siRNA chimera that showed promise in mouse models by interfering with the function of HER2 and EGFR receptors and inducing apoptosis in HER2-expressing cancer cells [102]. We have also developed a three-in-one AsiC, targeting EGFR, HER2, and HER3 in one molecule and significantly inhibited tumor growth in xenograft models [103]. These aptamers have yet to be explored in clinical trials due to lack of a viable delivery system into the target cells. However, with the nanotechnology now widely used to deliver COVID-19 spike protein mRNA into vaccines, it is now feasible to use the similar strategy to deliver aptamers against HER2 into HER2 + breast cancer.

Most disease-causing proteins are “undruggable” due to the lack of available binding pockets or suitable chemical matter. However, targeted protein degradation (TPD) is a novel therapeutic alternative that eradicates target proteins by utilizing endogenous protein degradation machineries. PROTACs (proteolysis-targeting chimeras) and molecular glues are two forms of TPD. PROTACs in particular are exciting because they appear to be suitable for oral administration and are less liable to resistance mutations [104]. This field is rapidly expanding and has shown promise in the field of breast cancer. A trastuzumab-PROTAC conjugate (Ab-PROTAC 3) was shown to induce catalytic protein degradation only in HER2 positive breast cancer cell lines, while sparing HER2-negative cells [105]. Finally, the emerging technology of molecular glue degrader makes it possible to target HER family proteins for degradation as well, including HER2 [106].

### Anti-HER2 immunotherapy

As with many malignancies, the role of immunotherapy is also being examined in HER2 + MBC. The KATE2 trial explored the use of atezolizumab with T-DM1 for treatment of HER2 + MBC that had progressed on trastuzumab and a taxane. Overall, the study was negative, but there may have been a benefit in patients who were PD-L1 positive [107]. This is similar to the results of other trials of immunotherapy in both HER2 + and triple negative MBC, in which the benefits of immunotherapy are generally restricted to PD-L1 positive patients [108–110]. Further study will be required to determine the role of immunotherapy in HER2 + MBC.

Immunotherapy via an anti-HER2 vaccine is an attractive concept because HER2 + breast cancer is one of the most immunogenic breast cancer subtypes. A vaccine would allow for continued active immune surveillance against the tumor, is cost-effective, and requires fewer administrations [111]. One of the most studied is the E75 vaccine (NP-S), which has been evaluated in a phase III clinical trial. E75 is an immunogenic peptide derived from the HER2 protein. The PRESENT trial enrolled patients with early-stage node-positive breast cancer with low-HER2 expression to either NP-S with GM-CSF or placebo monthly for 6 months. The study was stopped due to futility after 16 months. However, the possibility of pseudoprogression with the vaccine was raised by this study, as is seen with checkpoint inhibitor immunotherapy. Pseudoprogression occurs when immune infiltration of a cancer makes the tumor appear larger on imaging than it actually is. Recurrence of disease in this trial was determined by imaging only, and nearly three times more patients in the vaccine group had radiographic recurrence. Since the groups were well-balanced, it was unlikely that the vaccine itself caused the recurrence. Whether this phenomena is truly associated with the vaccine will be the work of future study [112] (Table 2).

**Table 2:** **Novel anti-HER2 therapy in development.** New anti-HER2 therapies that have entered clinical trials

Class of drug	Name	Phase of clinical trial	Clinical trial.gov number	Sponsor
TKI	Pyrotinib	3	NCT03080805	Jiangsu Hengrui Medicine
ADC	SYD985	3	NCT03262935	Byondis B.V
CAR-T	HER2 CAR T cells	1	NCT03696030	City of Hope Medical Center
Bispecific antibodies	ZW25	2	NCT04224272	Zymeworks
Bispecific T-cell engagers	PRS-343	1	NCT03330561	Pieris Pharmaceuticals
Biparatopic ADC	ZW49	1	NCT03821233	Zymeworks
PD-L1 inhibitor	Atezolizumab	2	NCT02924883	Hoffmann-La Roche
HER2 vaccine	Nelipepimut-S or E75	3	NCT01479244	Galena Biopharma

*TKI* tyrosine kinase inhibitor, *ADC* antibody–drug conjugate, *CAR-T* chimeric antigen receptor T-cell

## Conclusion

Metastatic HER2 + BC is a burgeoning field of development, with three new drugs approved in 2020 alone. In the late-line setting, it can be difficult to decide on the optimal drug sequencing for individual patients. After patients have progressed on docetaxel + trastuzumab + pertuzumab and T-DM1, we suggest choosing a TKI-based regimen based on the activity of TKIs in p95 and other forms of trastuzumab resistance as well as in brain metastases. Biomarkers are needed to help predict which therapies patients may benefit from the most, and ctDNA is promising. Numerous therapies are in development, which will be essential in improving survival in the future for patients with this aggressive disease.

## Data Availability

Not applicable.
